# Masquerading as Pneumonia: A Lung Neuroendocrine Tumor Case Report

**DOI:** 10.7759/cureus.46411

**Published:** 2023-10-03

**Authors:** Omeed S Jahangiri, Joshua R Robbins, Sivakumar Nagaraju

**Affiliations:** 1 Clinical Medicine/Internal Medicine, Burrell College of Osteopathic Medicine, Albuquerque, USA; 2 Internal Medicine, Burrell College of Osteopathic Medicine, Albuquerque, USA; 3 Pulmonary and Critical Care, Lovelace Medical Center, Albuquerque, USA

**Keywords:** cardiothoracic & vascular surgery, atelectasis, case report, suspicious lung mass, recurrent pneumonia, carcinoid tumors, tumor, internal medicine, pulmonology, lung neuroendocrine tumor

## Abstract

The presentation of recurrent pneumonia, particularly in the same lobe, should raise suspicion for possible neuroendocrine tumors of the lung within that respective lobe. Commonly, these types of tumors will have a gastrointestinal origin with a larger incidence of carcinoid syndrome, but they may also originate in the pancreas or lungs. This case illustrates the potential for a masked lung tumor in an otherwise young and healthy 31-year-old patient, with a short history of tobacco dependence and unremarkable family history, who presents with recurrent pneumonia and dyspnea. Although rare in itself, this case was even more unique due to the partial calcification of the neuroendocrine tumor mass along with causing a collapse in the entire right middle lobe.

## Introduction

Neuroendocrine tumors (NETs) are neoplasms that arise within neural cells and hormone-producing cells of the neuroendocrine system [1]. These tumors generally grow very slowly, taking years to progress, although they can sometimes grow rapidly.

NETs are most commonly found in the gastrointestinal (GI) tract (67.5% of cases), but the second most frequent location is the bronchopulmonary tract (25.3%) [2]. They occur more commonly in women, accounting for 52.7% of cases, and the incidence has increased more than sixfold since 1973 [3]. Tumors in the lung arise from the Kulchitsky cells of the bronchial mucosa [4].

Bronchial NETs typically originate in the proximal airways and can produce symptoms such as airway obstruction or bleeding. Common clinical symptoms include wheezing, dyspnea, cough, chest pain, and hemoptysis, as well as recurrent pneumonia in the lobe where the tumor is located. These symptoms are not specific to NETs but are generally associated with lung masses.

Lung NETs have a lower incidence of carcinoid syndrome compared to small bowel NETs because they typically release serotonin and other vasoactive substances in smaller quantities [5]. Clinical manifestations of carcinoid syndrome in lung NETs are similar to those in other primary NETs and include facial flushing, shortness of breath, high blood pressure, diarrhea, weight gain, and hirsutism [6]. These cases, categorized as functional NETs, will show increased urinary levels of 5-hydroxyindoleacetic acid (5-HIAA). Conversely, non-functional NETs, which lack any signs or symptoms, are usually diagnosed at a later stage.

Furthermore, they can be classified as either typical or atypical. Atypical NETs have been known to be accompanied by calcification or ossification, and the presentation of a single tumor nodule with large ossification is rare. Typical carcinoids are generally less aggressive than atypical ones, although they appear similar both radiologically and pathologically [7].

Lung NETs account for 1 to 2% of all pulmonary neoplasms and over 25% of all carcinoid tumors. Approximately 10 to 20% of pulmonary carcinoids are atypical, leaving the remaining 80 to 90% as typical carcinoids [7]. The mechanisms by which this disease is acquired are not well understood. However, factors that may increase an individual's risk include inherited syndromes such as Multiple Endocrine Neoplasia type 1 (MEN1), Von Hippel-Lindau, Neurofibromatosis type 1 (NF-1), Tuberous Sclerosis Complex (TSC), and Multiple Endocrine Neoplasia type 2 (MEN2) [8].

Diagnosis generally stems from clinical signs and imaging but is strongly confirmed through biopsy. On CT imaging, carcinoid tumors usually appear as ovoid or spherical nodules with well-defined lobulated borders. They may also appear elongated along a parallel axis with adjacent bronchi [7].

Therapies can include medications like somatostatin analogs, peptide receptor radionuclide therapy, or targeted therapy. However, surgery has been shown to be the most successful and well-tolerated treatment. A somatostatin analog, such as octreotide, inhibits various hormones and peptides like serotonin, gastrin, and vasoactive intestinal peptide, thereby providing relief from carcinoid syndrome symptoms [9].

The purpose of this case study is to highlight the importance of considering NETs when diagnosing recurrent pneumonia. Further testing should also be conducted following such diagnoses to identify the underlying causes, benefiting both the patient and the broader understanding of the disease's pathogenesis.

## Case presentation

**Figure 1:**
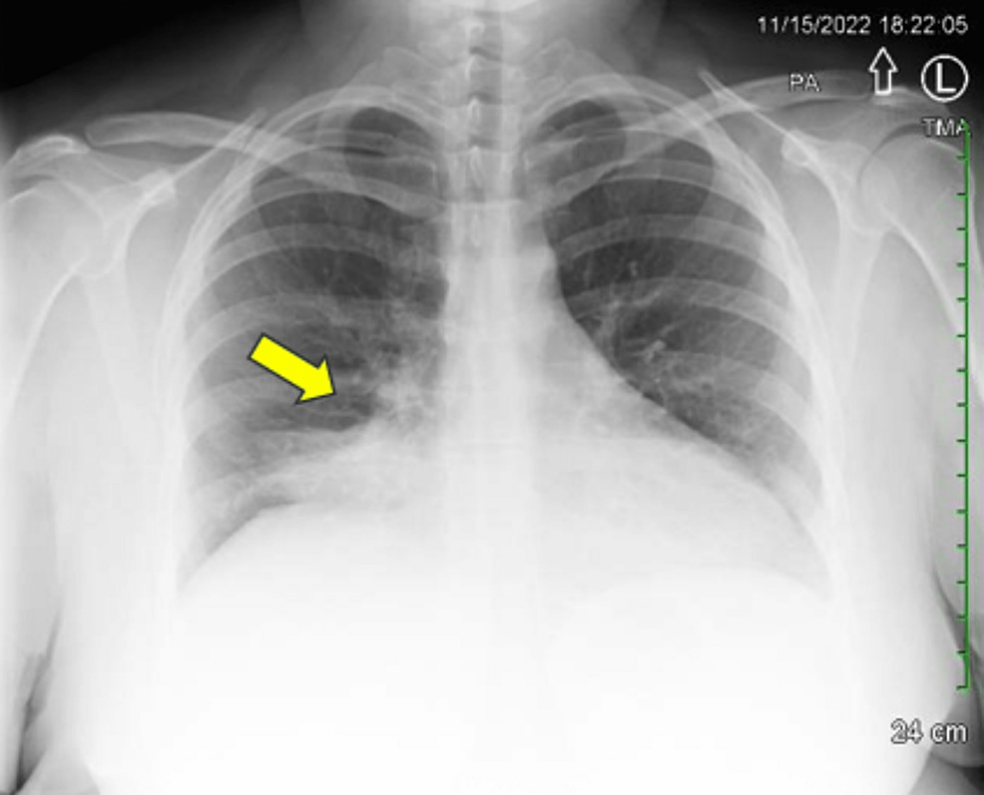
Posterior/anterior chest X-ray from November 2022. The yellow arrow points toward the tumor region.

A 31-year-old female patient, whose past medical history was only significant for obesity (with a BMI of ~42) and tobacco dependence (2 pack-years and currently smoking 0.25 packs per day), initially presented to the emergency department at our hospital in November 2022. She was evaluated for five days of dry cough, accompanied by shortness of breath upon exertion. At the same time, her husband had also reportedly been experiencing similar symptoms. Testing for SARS-CoV-2 (COVID-19) and Influenza A+B returned undetectable results. Procalcitonin levels were not measured at this time. The initial chest X-ray, depicted in Figure 1, showed evidence of focal consolidation at the base of the right lung, raising concerns for pneumonia. Computed tomography (CT) imaging was recommended if clinically warranted; however, it was not ordered at that time. The patient was ultimately discharged with a prescription for doxycycline.

**Figure 2:**
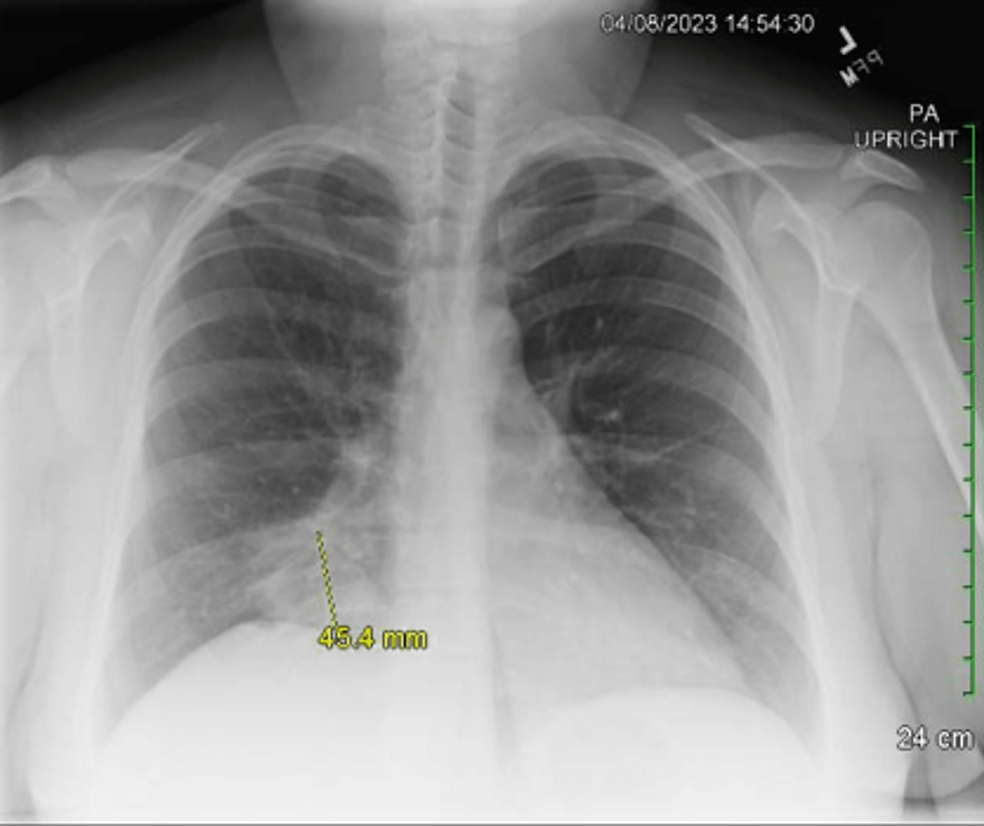
Posterior/anterior chest X-ray from April 2023. The yellow radiology marker depicts the area of interest as well as the marked size.

**Figure 3:**
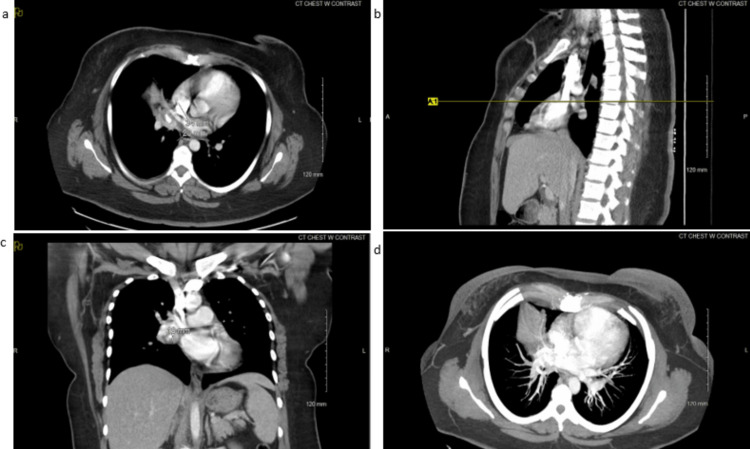
CT imaging of chest and lung from April 2023. (a) Mid-axial CT of the chest. The white radiological markings indicate the tumor area size. (b) Saggital CT of the chest. The yellow radiological marker indicates the tumor area of interest. (c) Coronal CT of the chest. The white radiological marker indicates the tumor area size. (d) Axial maximum intensity projection (MIP) CT of the lungs.

The patient returned to the emergency department at our hospital in April 2023 for evaluation of a three-day history of worsening dry cough, shortness of breath, sore throat, body aches, and chills. Her symptoms were similar to, but more severe than, her previous episode in November 2022. Initial vital signs indicated a temperature of 100.4°F, a heart rate of 133 bpm, an oxygen saturation of 95%, and a blood pressure of 166/93 mmHg. Complete blood count (CBC) and complete metabolic panel (CMP) were significant only for a raised white blood cell count (WBC) of 14.9 k/µL. Troponin levels were less than 0.017 ng/mL. Initial procalcitonin was 0.12 ng/mL and later decreased to 0.9 ng/mL. Molecular tests for Group A Streptococcus, Influenza A+B, and SARS-CoV-2 were all negative. A chest X-ray again revealed evidence of consolidation in the right lobe. Subsequent chest CT imaging identified a 3.4 cm partially calcified right inferior hilar mass extending into, and occluding, the right middle lobe bronchi, along with post-obstructive atelectasis and collapse of the middle lobe. Images from this visit are depicted in Figures 2, 3. Consideration for bronchoscopy and sampling was recommended for further diagnostic assessment. While in the Emergency Department, she was administered a 3.375 g intravenous dose of piperacillin-tazobactam and subsequently admitted to the hospital for pulmonology follow-up on hilar adenopathy and atelectasis. Due to increasing dyspnea and an oxygen saturation level of 88% upon admission, she was placed on two liters of supplemental oxygen via nasal cannula.

 

Upon pulmonology consultation, the patient denied owning pets associated with pulmonary issues, having concerns about mold presence, or having large amounts of wall-to-wall carpeting at home or work. She also denied any occupational exposures, such as mining, farming, asbestos, or animal exposure. She had not received prior vaccinations for COVID-19 or influenza, but reported a previous COVID-19 infection that resolved without medical intervention. The pulmonology team evaluated for granulomatous lung disease, testing for sarcoidosis, coccidioidomycosis titers, and QuantiFERON, and monitored procalcitonin levels. All titers were negative. Procalcitonin was 0.15 ng/mL and remained stable. Sarcoidosis was also considered in the differential diagnosis at this time. An Endoscopic Bronchial Ultrasound (EBUS) with bronchoalveolar lavage and hilar lymph node biopsy was planned. The patient continued on a regimen of ampicillin/sulfabactam 3g IV every 6 hours and doxycycline 100 mg orally. Her symptoms improved substantially during her admission, although a dry cough and shortness of breath persisted.

EBUS showed evidence of endobronchial obstruction in the right middle lobe with patent right lower lobe subsegments, along with the involvement of the right middle lobe carina and mucosa below. Biopsies were obtained. During and after the EBUS procedure, she was administered tranexamic acid, a dose of dexamethasone, and racemic epinephrine, which led her to experience respiratory distress. Consequently, she was transferred to the ICU and placed on non-invasive ventilation via bilateral positive airway pressure (BiPAP). Nebulizer treatments with ipratropium 0.5 mg/2.5 ml and albuterol 2.5 mg/0.5 ml were ordered four times daily as needed. The patient was determined to require home oxygen, as her room air oxygen saturation was 85% at rest and remained at 85% with ambulation (home oxygen was administered via nasal cannula at 2 LPM continuously to achieve an O2 saturation of 90% with ambulation). Biopsy results showed that the cells of interest were strongly positive for pancytokeratin, synaptophysin, and chromogranin. Thyroid transcription factor-1 (TTF-1) was negative, and the Ki-67 human marker was difficult to quantify on the disaggregated aspirate but appeared to comprise less than 2% of cells. Cluster of differentiation protein (CD45) highlighted scattered inflammatory cells. According to the laboratory, these findings were consistent with well-differentiated NETs (carcinoid). The final grading was deferred pending the possibility of a solid tissue biopsy or resection but was thought to be low grade. Immunohistochemistry testing was developed and its performance was determined by a local laboratory's histology department. She was discharged on the fourth day of admission with prescriptions for an albuterol rescue inhaler, amoxicillin-clavulanate 875-125 mg orally twice daily for seven days, benzoate 100 mg orally three times daily for seven days, and Varenicline 1 mg orally for her nicotine dependence.

The patient was then re-evaluated in the pulmonology clinic about 14 days later. She felt much improved but would still experience mild dyspnea with exertion. Pulmonary function tests were conducted and the results are presented in Table 1. Consultations with cardiothoracic surgery and oncology were arranged for further management of her NET.

**Table 1:** Pulmonary Function Testing, June 2023 Abnormal laboratory values are marked as ***. Normal reference ranges are alongside each abnormal laboratory value descriptor. FEV10: forced expiratory volume after 10 seconds, FEV1: forced expiratory volume after 1 second, FVC: forced vital capacity, DLCO: diffusion capacity of the lungs for carbon monoxide, RV: residual volume, TLC: total lung capacity, VC: vital capacity, Pre: before bronchodilator, post: after bronchodilator.

Component	Value	Component	Value
Post-FEV10 (L)	3.61	Pre-FVC (%)	4.37 or 120
Post-FEV1 (%)	117	Pre-FEV1 (%)	112
Post-FVC (%)	118	Pre-RV (L)***	0.71*** (0.75-1.20)
Pre-DLCO (mL/min mmHg)	22.81	Pre-TLC (L)	5.09
Pre-FEV10 (L)	3.45	Pre-VC (L)	4.38

Upon evaluation by the cardiothoracic surgery team in early May 2023, the patient still had a productive cough accompanied by mild cold-like symptoms, but otherwise felt well and had no other acute complaints. It was concluded that surgical intervention would be the most beneficial course of action for her; no other specific pharmacological treatments were initiated due to her health insurance status at the time of her initial consultations. The oncology team also recommended surgery as the best course of action, and the patient agreed. She was scheduled for a right thoracotomy and bi-lobectomy (involving the right middle and right lower lobes) with a frozen section analysis. An echocardiogram was ordered for pre-surgical cardiac clearance and ultimately showed normal results.

In August 2023, the patient underwent a preoperative bronchoscopy that revealed the tumor to be more extensive than initially believed. As a result, the originally planned surgery was canceled with the intention to reschedule. The cardiothoracic surgery team now anticipates that the patient will require either a pneumonectomy or a hand-sewn anastomosis, scheduled for October 2023. Further staging of the tumor has not yet been completed.

## Discussion

As previously mentioned, one clinical feature that can delay the diagnosis of a lung neuroepithelial tumor is recurrent pneumonia in the same lobe as the tumor. This is one potential reason why patients can undergo several courses of antibiotics before the true cause of their presentation is known. Initial diagnostic imaging is usually conducted via a contrast-enhanced CT scan of the chest to determine the tumor's location, extent, and the presence or absence of mediastinal lymphadenopathy. Contrast-enhanced CT is generally considered superior to X-ray because tumors commonly have lobulated borders and calcifications; CT imaging causes a lower level of distortion on the image [10]. If a lung NE tumor is suspected, neuroendocrine markers such as chromogranin and synaptophysin can be used to help distinguish the type of lung neoplasm [11]. Increased 5-hydroxyindoleacetic acid on a 24-hour urine screen can be a helpful initial biochemical test for carcinoid tumors. Positron Emission Tomography (PET-CT) is generally the preferred imaging technique for lung tumor metastasis detection [12]. Oftentimes, initial imaging can be accompanied by somatostatin receptor-based imaging techniques. It is recommended that a baseline somatostatin receptor-based imaging technique is done alongside routine cross-sectional imaging because this can be a predictor of clinical response to therapies with somatostatin analogs, such as octreotide [13]. After a tumor is identified, definitive diagnosis is best done with bronchoscopy and biopsy with prompt cardiothoracic surgery consultation. Pulmonary function testing is warranted in these patients but does not have a defined pattern of presentation with the values; our patient's lower residual volume follows her known atelectasis of the right middle lobe/obstruction of the lobe. If air cannot enter the lungs normally, the volume of air left over in the lungs after expiration will logically be lower as well. Somatostatin analogs or antiproliferative agents can be used as options to help slow disease progression for carcinoid syndrome. Ultimately, the definitive treatment for the large majority of lung NETs is surgical resection with further resection of metastasis whenever possible for curative intent [14]. The underlying factors, whether biological or environmental, resulting in higher incidence are still unknown [15]. Better access to healthcare, improved technology, and higher incidence of electronic cigarette use in more recent times may suggest causes for increased incidence, but further studies should be done to confirm these thoughts. According to the existing literature on lung carcinoid tumors, the average 5-year survival rate for typical NETs is 93% (range 88-97%), and for atypical NETs, it is 69% (range 40-86%) [16].

Lastly, ossification is usually only found in patients with pulmonary NETs and is seen in up to 10% of cases, particularly those of longer duration. In bone carcinoid tumors, an osteoblastic reaction occurs around the tumor cells in the NETs. It is possible that the ossification is initiated by factors secreted by the tumor cells, which then act on the surrounding tissue. A proposed mechanism is the production of osteogenic factors, such as bone morphogenic protein-2 (BMP-2), facilitated by the surrounding tissue [17-18]. BMP-2 induces osteoblastic differentiation of immature osteoblasts via activation of transcription factors and other molecular switches that regulate bone development [18-20].

## Conclusions

Most clinicians are well-versed in diagnosing pneumonia, and even to some extent, lung tumors in general. However, when these two conditions present together, the underlying tumor can be masked. When a patient presents with recurrent pneumonia, further workup is worth considering, such as a 24-hour urine 5-hydroxyindoleacetic acid screen, BMP-2, chromogranin, or synaptophysin to help guide clinical suspicion. If a tumor is identified, referral for cardiothoracic surgery evaluation is indicated, and treatment is typically carried out via resection. Somatostatin analogs may also be added as antiproliferative agents and for symptomatic control. Although this patient was young and otherwise healthy with no prior family history, the importance of this case is underscored by the fact that it can still occur with no clear preceding factors. Although rare in itself, this case was unique both in clinical presentation and due to the calcification of the NET mass, which caused a collapse in the entire right middle lobe. Clinical suspicion should be warranted in the presentation of patients with similar respiratory signs and symptoms.
